# Preventing heat illness in the anticipated hot climate of the Tokyo 2020 Summer Olympic Games

**DOI:** 10.1186/s12199-017-0675-y

**Published:** 2017-09-19

**Authors:** Takeyasu Kakamu, Koji Wada, Derek R. Smith, Shota Endo, Tetsuhito Fukushima

**Affiliations:** 1 Department of Hygiene and Preventive Medicine, School of Medicine, Fukushima Medical University, Hikarigaoka 1, Fukushima, 960-1295 Japan; 2 Bureau of International Health Cooperation, National Center for Global Health and Medicine, 1-21-1 Toyama, Shinjuku-ku, Tokyo, 162-8655 Japan; 3 College of Public Health, Medical and Veterinary Sciences, James Cook University, Townsville, Australia

## Abstract

Amid the effects of global warming, Tokyo has become an increasingly hot city, especially during the summertime. To prepare for the upcoming 2020 Summer Olympics and Paralympics in Tokyo, all participants, including the athletes, staff, and spectators, will need to familiarize themselves with Tokyo’s hot and humid summer conditions. This paper uses the wet-bulb globe temperature (WBGT) index, which estimates the risk of heat illness, to compare climate conditions of sports events in Tokyo with the conditions of the past three Summer Olympics (held in Rio de Janeiro, London, and Beijing) and to subsequently detail the need for establishing appropriate countermeasures. We compared WBGT results from the past three Summer Olympics with the same time periods in Tokyo during 2016. There was almost no time zone where a low risk of heat illness could be expected during the time frame of the upcoming 2020 Tokyo Olympics. We also found that Tokyo had a higher WBGT than any of those previous host cities and is poorly suited for outdoor sporting events. Combined efforts by the official organizers, government, various related organizations, and the participants will be necessary to deal with these challenging conditions and to allow athletes to perform their best, as well as to prevent heat illnesses among staff and spectators. The sporting committees, as well as the Olympic organizing committee, should consider WBGT measurements in determining the venues and timing of the events to better avoid heat illness and facilitate maximum athletic performance.

## Background

Amid the effects of global warming, heat-related health risks are increasing [1] especially in sporting events [2, 3]. Global warming is believed to be an ongoing phenomenon, and thermal environment management, particularly heat stress, is growing in importance. Caution is therefore needed to help guard against these health risks during summer sporting events [4]. The 2016 Summer Olympics held in the tropical city of Rio de Janeiro, for example, faced potential environment conditions of 19.6 to 28.7 °C and ~ 71% relative humidity [5]. The high visibility of international athletic events such as the Summer Olympics represents just a small fraction of the intense physical exertion undertaken outdoors, and increasing restrictions on when, where, and how the Games can be held in consideration of extreme heat conditions suggest the growing magnitude of this issue [6]. On the other hand, heat illness in athletes is considered to be preventable and countermeasures are necessary for team staffs [7].

Tokyo will host the Summer Olympics from 24 July to 9 August 2020 [8]. Tokyo is the world’s largest metropolitan area [9], and the effects of this vast urbanization further aggravate the damp thermal environment [10]. Recently, the total number of individuals suffering from heat illness is rising in Tokyo [1]. Interestingly, the previous Tokyo Summer Olympics of 1964 were actually held in autumn (10–24 October), and therefore, the upcoming 2020 event will present even greater heat-related challenges.

The most common recommendation to currently follow is that sporting event organizers reschedule or cancel an event based on the wet-bulb globe temperature (WBGT), an index based on a combination of ambient temperature, relative humidity, the heat-load impact of solar radiation, and the cooling power of wind [4, 11]. WBGT is a validated empirical index of environmental heat stress and has been awarded an ISO (ISO/DIS 7933 1984) certification [12, 13]. WBGT was initially developed by the US military [14] in recognition that heat illness is caused by the combined effects of high temperatures, high humidity, and solar radiation on the human body [15, 16]. Research utilizing the WBGT also focused on developing safe limits for physical exertion in hot environments [17]. The index later became popular in sports medicine via the American College of Sports Medicine (ACSM) (Table 1) [15] and was subsequently adopted by various other sporting federations. The International Tennis Federation (ITF), for instance, uses it to decide on the suspension of play, while the Federation of International Football Association (FIFA) uses it to determine breaks for players to cool down [18, 19].

**Table 1:** Wet-bulb globe temperature (WBGT) levels for modification or cancelation of workouts or competition for healthy adults [15]

WBGT		Continuous activity and competition	Training and non-continuous activity	
°F	°C		Non-acclimatized, unfit, high-risk individuals^a^	Acclimatized, fit, low-risk individuals^a,b^
≤ 50.0	≤ 10.0	Generally safe; EHS can occur associated with individual factors	Normal activity	Normal activity
50.1–65.0	10.1–18.3	Generally safe; EHS can occur	Normal activity	Normal activity
65.1–72.0	18.4–22.2	Risk of EHS and heat illness begins to rise; high-risk individuals should be monitored or not compete	Increase the rest: work ratio. Monitor fluid intake.	Normal activity
72.1–78.0	22.3–25.6	Risk for all competitors is increased	Increase the rest: work ratio; decrease total duration of activity.	Normal activity monitor fluid intake.
78.1–82.0	25.7–27.8	Risk for unfit, non-acclimatized individuals is high	Increase the rest to work ratio; decrease intensity and total duration of activity.	Normal activity monitor fluid intake.
82.1–86.0	27.9–30.0	Cancel level for EHS risk	Increase the rest to work ratio to 1:1, decrease intensity and total duration of activity. Limit intense exercise. Watch at-risk individuals carefully.	Plan intense or prolonged exercise with discretion^c^, watch at-risk individuals carefully
86.1–90.0	30.1–32.2		Cancel or stop practice and competition.	Limit intense exercise^c^ and total daily exposure to heat and humidity; watch for early signs and symptoms
≥90.1	≥32.3		Cancel exercise.	Cancel exercise uncompensable heat stress^d^ exists for all athletes^c^

*EHS* exertional heat stroke, *WBGT* wet-bulb globe temperature

^a^While wearing shorts, T-shirt, sock, and sneakers

^b^Acclimatized to training in the heat at least 3 week

^c^Differences of local climate and individual heat acclimatization status may allow activity at higher levels than outlined in the table, but athletes and coaches should consult with sports medicine staff and should be cautions when exceeding these limits

^d^Internal heat production exceeds heat loss and core body temperature rises continuously, without a plateau

Several studies have estimated potential WBGT values expected during the 2020 Tokyo Olympics and have suggested a time shift for events [20, 21]. However, the temperature of Tokyo is high even at midnight, so it is unknown whether such a time shift would be effective. The present study aimed to compare the WBGT measurements by time zone in Tokyo with those of the past three Summer Olympics and to detail the need for establishing appropriate countermeasures for the upcoming 2020 Olympics in Tokyo.

## Method of assessment of WBGT index for recent Summer Olympics and Tokyo

We referred to WBGT measurements for the host cities of the past three Summer Olympics (in Rio de Janeiro, London, and Beijing). Although WBGT and global temperatures are not always maintained in weather records, Australia’s Bureau of Meteorology approximates it from air temperature and relative humidity as follows [12].

The simplified formula is:

WBGT = 0.567 × Ta + 0.393 × (rh/100 × 6.105 × exp (17.27 × Ta/(237.7 + Ta))) + 3.94

where:

Ta = air (dry − bulb) temperature (^°^C)

rh = relative humidity (%)

The formula assumes a moderately high radiation level in light wind conditions. We note that the calculated WBGT can estimate daytime conditions accurately but may overestimate nighttime and early morning conditions [12].

Temperature and humidity data for every 3 h on event dates are recorded on the World Weather Online website [22]. To estimate and compare with past host cities, we used the past 10 years (2008–2017) of data for the same dates in Tokyo (the place of the new Tokyo Olympic Stadium). WBGT measurements at 00:00, 03:00, 06:00, 09:00, 12:00, 15:00, 18:00, and 21:00 were utilized.

## Comparison of WBGT at previous three Summer Olympics with current summer climate in Tokyo

Table 2 shows the median and 25th–75th percentile WBGT by time of day for each city. Tokyo recorded the highest WBGT at each time, and the median WBGT was at a “Cancel level for EHS risk for continuous activity and competition” at all times. From 09:00 to 18:00, the median WBGT was at “cancel exercise uncompensable heat stress exists for all athletes” (33, 34, 34, and 33 °C at 09:00, 12:00, 15:00, and 18:00, respectively). In fact, none of the measured WBGT predicted a low risk of heat illness. These data suggest that Tokyo’s climate poses an extremely high risk for outdoor sports in the summer.

**Table 2:** Wet-bulb globe temperature (WBGT) by time of day in the previous three Summer Olympics and the expected periods of the Tokyo 2020 Summer Olympics

	00:00	03:00	06:00	09:00	12:00	15:00	18:00	21:00
Tokyo	30 (29–32)	30 (28–31)	30 (28–31)	33 (31–35)	34 (32–36)	34 (32–36)	33 (31–34)	31 (30–33)
Rio de Janeiro	23 (22–24)	23 (21–23)	22 (20–23)	26 (25–28)	31 (27–32)	30 (26–31)	29 (25–31)	26 (24–27)
London	18 (16–19)	17 (15–18)	18 (16–19)	21 (19–22)	22 (21–24)	22 (21–24)	21 (21–24)	19 (18–20)
Beijing	21 (20–25)	21 (20–24)	24 (22–27)	29 (27–31)	31 (30–32)	31 (30–32)	27 (26–28)	24 (22–25)

Median (25th–75th percentile) WBGT (°C) Tokyo: 24 July to 9 August from 2008 to 2017 (dates scheduled for Tokyo 2020 Summer Olympics) Rio de Janeiro: 5–21 August 2016 London: 25 July–12 August 2012 Beijing: 8–24 August 2008

**Fig. 1:**
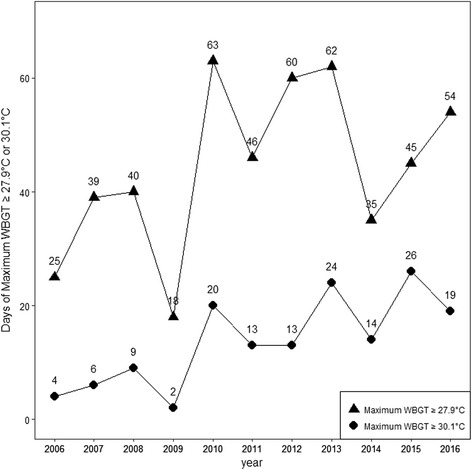
Days where the maximum WBGT (wet-bulb globe temperature) index was “Cancel level for EHS risk for continuous activity and competition” (WBGT ≥ 27.9 °C), “very high risk,” and “cancel or stop practice and competition” (WBGT ≥ 30.1 °C) in Tokyo from 2006 to 2016, as per the American College of Sports Medicine criteria [15]

We plotted days when the maximum WBGT was ≥ 27.9 °C (“cancel level for EHS risk for continuous activity and competition” as per the ACMS classification) and ≥ 30.1 °C (“cancel or stop practice and competition” per ACMS classification) as reported in data from the Ministry of the Environment, Japan (Fig. 1) [21]. In 2010, the days of maximum WBGT in Tokyo ≥ 27.9 or 30.1 °C showed an increase and have maintained a high level in each summer (July–September) since then. These data, therefore, suggest the risk of heat illness is also increasing in recent years. Not only for outdoor events but also indoor sporting events present a risk of heat illness during the daytime because spectators can be expected to move around both inside and outside the venue.

## Desirable preventive measures from the sports organizing committees

We determined that the WBGT during the Tokyo 2020 Olympics would likely be classified as high risk to a point where stoppage of play would be recommended at almost any time. Establishing and implementing effective countermeasures against heat illness is a vital task in preparations for the event. These measurements must be led by the official organizers, government, various related organizations, and the participants who will also need to take a number of preparatory measures and to establish an appropriate emergency medical system during the upcoming Olympic Games.

In cooperation with individual sporting associations, WBGT scores should be used as a standard when creating guidelines for measures taken to prevent heat illness. The Japanese Ministry of the Environment, for example, has published heat illness guidelines for summer events for event organizers and facility managers [23]. WBGT scores around and in venues should be considered in determining which venues are suitable. In addition, the effect of heat stress can also differ between individuals, and therefore, it is necessary to take measures according to their attributes such as athletes, staff, and spectators. Heat illness involves a complex interaction between environmental heat strain, clothing, and human thermal physiology [11]. Preventive measures should therefore consider various strategies and management protocols specifically targeting environmental heat stress.

The ACSM has declared that all summer events should be scheduled for the early morning, ideally before 08:00, or in the evening after 18:00, to minimize solar radiation [15]. In Tokyo, for almost all daytime hours during equivalent event dates in 2016, the WBGT records demonstrated levels of “Cancel level for EHS risk for continuous activity and competition” or “cancel or stop practice and competition,” and therefore, even conducting these events in the early morning and nighttime might not be sufficient to avert heat illness. As heat illness is also related to the exercise intensity and duration, desirable schedules must also be matched to individual sports.

Acclimatization to heat can be critical in preventing heat illness, as it increases tolerance, adjusts the body’s sweating mechanism (such as the threshold body temperature for sweating), causes excessive sweating, and decreases the amount of sodium lost through sweat [24]. Recently, heat acclimatization before competing in the heat has become an important measure for athletes [4, 25], and heat acclimation has also become part of the training regimen for sports competition [24, 25]. The risk of heat illness among athletes seems to be not so high despite the high WBGT index [26]. In 2015, the International Association of Athletics Federations (IAAF) World Athletics Championships in Beijing, China, only 15% of athletes were heat acclimatized even though hot and humid conditions were expected [27]. Differences of local climate and individual heat acclimatization status may allow activity at higher levels than outlined in the table, but athletes and coaches should consult with sports medicine staff and should remain cautious when exceeding these limits. Given that spectators and staff at the Olympic Games can probably not be expected to acclimatize to the heat, the organizing committee should also consider appropriate measures for non-acclimatized individuals.

Organizing committees can provide heat acclimation programs for staff. The National Athletic Trainers’ Association in the USA, for example, has determined heat acclimatization guidelines for secondary school athletics [28]. In those guidelines, activity time is restricted during the initial exertion period. For non-acclimatized workers, outdoor working times should be limited during this time period. The appropriate selection and design of staff uniforms is also important to help reduce heat strain. These uniforms should be adapted to the heat and be wearable by all staff, while break times should be frequently provided so that staff can hydrate frequently.

For spectators, especially those from climates unlike Tokyo’s, the organizing committee should broadly disseminate information and warnings on the importance of taking measures to help prevent heat illness. These include maintaining physical health by means such as getting enough sleep and nourishment, wearing sun-shading implements such as wide-brimmed hats, and drinking sufficient amounts of water throughout the day.

The Tokyo Olympic organizing committee clearly needs to be mindful of the risk of heat strain when considering how to organize and schedule outdoor events. The adoption of combined and coordinated methods in accordance with the prevailing conditions will help lead the way to effective countermeasures. Organizing committees in Japan as elsewhere should pursue combined countermeasures when dealing with challenging thermal environments such as arranging shaded areas and mist showers and distributing water and/or sun-shading implements, similar to those protocols adopted during the 1996 Atlanta Summer Olympics [29]. In addition, since primary care is important for heat illness, an effective emergency medical care system needs to be implemented for the Tokyo Olympic Games. Such systems will need to be accessible, affordable, and multilingual so as to offer the greatest possible benefit to the widest range of individuals.

This study has some potential limitations that should be noted. First, WBGT has been recently challenged [4, 30], and various new tools, models, and indices for the assessment of human exposures to heat have now been developed [11]. Second, we calculated WBGT from available air temperature and humidity records, whereas WBGT would usually be measured by three thermometers—a natural wet bulb, a globe, and sometimes a dry bulb [12]. Despite this, the WBGT remains a widely known and useful index for helping to predict the risk of heat illness [4]. Third, although WBGT has been awarded an ISO (ISO/DIS 7933 1984) certification [12, 13] and Japan Industrial Standard (JIS) Z8504, many kinds of WBGT index meters are used. Japanese Industrial Standards Committee, for example, has recently published an electronic WBGT index meter as JIS B7922 in March 2017 [31]. WBGT should be measured by equipment which conforms to JIS Z8504 or JIS B7922. Given that it is still necessary to take measures according to the individual attributes of athletes and other participants, the use of various indicators and their applications should be expanded in order to more accurately assess the risks of heat illness.

## Conclusion

Overall, our study suggests that the Tokyo 2020 Summer Olympics will be held amid extremely high WBGT conditions, including at levels deemed poorly suited for conducting sporting events. Combined efforts by all stakeholders during these events will therefore be necessary to deal with these challenging conditions so that athletes can perform their best and so heat illness can be minimized among individuals taking part in these activities. Sporting committees and the Olympic organizing committee should also consider WBGT in selecting venues and the timing of events to help minimize heat illness and enable maximum performance by athletes. Similarly, the organization of the 2020 Tokyo Olympics will need to manage heat as an occupational safety issue for staff and also provide multiple solutions to help heat illness among spectators and tourists.

## Abbreviations

ACSM: American College of Sports Medicine
FIFA: Federation of International Football Association
IAAF: International Association of Athletics Federations
ITF: International Tennis Federation
JIS: Japan Industrial Standard
WBGT: Wet-bulb globe temperature
